# THE ROLE OF CAPRIN-1 PROTEIN DYSREGULATION IN SYNAPSE DECLINE LEADING TO PROGRESSION OF TAUOPATHIES

**DOI:** 10.1093/geroni/igz038.3078

**Published:** 2019-11-08

**Authors:** Caitlyn Fastenau, Helen Cifuentes, Grant Kauwe, Tara Tracy

**Affiliations:** 1 University of California, Davis, Davis, California, United States; 2 Buck Institute for Research on Aging, Novato, California, United States

## Abstract

Many neurodegenerative diseases are characterized by accumulation of proteins such as tau, a microtubule stabilization protein. Toxic tau forms tangles and affects neuronal synapse function, an early step of neurodegeneration. To focus on synapse function, we highlighted Caprin-1 protein. Through gene ontology, Caprin-1 is related to RNA granule proteins, important for transport and local translation in dendrites. Caprin-1 is of interest for neurodegeneration because it is a memory related protein, transports mRNA in RNA granules, and knock out mice demonstrate memory deficits. As we age, the performance of local translation in the dendrites is compromised and leads to synapse dysfunction. We hypothesize Caprin-1 binds to Tau and becomes disrupted. This leads to the dissolution of RNA granules, inhibition of mRNA transport in dendrites, suppression of translation, and failure of synapse. Early western blot data showed reduced Caprin-1 in PS19 Tau+, supporting our model that Caprin-1 is disrupted in disease models. Through immunohistochemistry, we investigated the localization of Caprin-1 in the mouse hippocampus. We observed Caprin-1 localization to dendrites of CA1 neurons in the hippocampus. Furthermore, Caprin-1 exhibited colocalization with Rps6, an RNA granule marker. This suggests Caprin-1 associates with RNA granules in mouse hippocampus. Finally, we investigated the localization of Caprin-1 in human iPSC-derived neurons. Similar to the mouse hippocampus, we observed localization of Caprin-1 to dendrites of human neurons. In future directions, we will examine whether pathogenic tau alters the association of Caprin-1 with RNA granules and the mechanisms by which pathogenic tau negatively effects synapse function.

